# Switching from one biologic to benralizumab in patients with severe eosinophilic asthma: An ANANKE study *post hoc* analysis

**DOI:** 10.3389/fmed.2022.950883

**Published:** 2022-09-02

**Authors:** Cristiano Caruso, Paolo Cameli, Elena Altieri, Maria Aliani, Pietro Bracciale, Luisa Brussino, Maria Filomena Caiaffa, Giorgio Walter Canonica, Stefano Centanni, Maria D’Amato, Stefano Del Giacco, Fausto De Michele, Elide Anna Pastorello, Girolamo Pelaia, Paola Rogliani, Micaela Romagnoli, Pietro Schino, Marco Caminati, Alessandra Vultaggio, Alessandro Zullo, Sara Rizzoli, Silvia Boarino, Gianfranco Vitiello, Francesco Menzella, Fabiano Di Marco

**Affiliations:** ^1^Dipartimento di Scienze Mediche e Chirurgiche, Fondazione Policlinico A. Gemelli, IRCCS, Università Cattolica del Sacro Cuore, Rome, Italy; ^2^Respiratory Diseases and Lung Transplantation, Department of Medical and Surgical Sciences and Neurosciences, Siena University Hospital, Siena, Italy; ^3^Reparto di Pneumologia, P.O. Garbagnate Milanese, Garbagnate Milanese, MI, Italy; ^4^UO Pneumologia e Pneumologia Riabilitativa, ICS Maugeri, IRCCS Bari, Bari, Italy; ^5^Reparto di Pneumologia, Ospedale Ostuni, Ostuni, BR, Italy; ^6^Dipartimento di Scienze Mediche, SSDDU Allergologia e Immunologia Clinica, Università degli Studi di Torino, AO Ordine Mauriziano Umberto I - Torino, Turin, Italy; ^7^Cattedra e Scuola di Allergologia e Immunologia Clinica, Dipartimento di Scienze Mediche, Università di Foggia, Foggia, Italy; ^8^Department of Biomedical Sciences, Humanitas University, Pieve Emanuele, MI, Italy; ^9^Asthma and Allergy Unit, IRCCS Humanitas Research Hospital, Rozzano, MI, Italy; ^10^Respiratory Unit, ASST Santi Paolo e Carlo, Department of Health Sciences, Università degli Studi di Milano, Milan, Italy; ^11^UOSD Malattie Respiratorie “Federico II,” Ospedale Monaldi, AO Dei Colli, Naples, Italy; ^12^Department of Medical Sciences and Public Health, University of Cagliari, Cagliari, Italy; ^13^UOC Pneumologia e Fisiopatologia Respiratoria, AORN A. Cardarelli, Naples, Italy; ^14^Allergy and Immunology, Niguarda Hospital, Milan, Italy; ^15^Dipartimento di Scienze della Salute, Università Magna Graecia, Catanzaro, Italy; ^16^Division of Respiratory Medicine, University Hospital “Tor Vergata,” Rome, Italy; ^17^Unit of Respiratory Medicine, Department of Experimental Medicine, University of Rome “Tor Vergata,” Rome, Italy; ^18^UOC Pneumologia, ULSS 2 Marca Trevigiana, Treviso, Italy; ^19^Fisiopatologia Respiratoria, Ospedale Generale Regionale, Ente Ecclesiastico “F. Miulli,” Acquaviva delle Fonti, BA, Italy; ^20^Department of Medicine, University of Verona, Verona, Italy; ^21^Allergy Unit and Asthma Center, Verona University Hospital, Verona, Italy; ^22^Immunoallergology Unit, Careggi University Hospital, Florence, Italy; ^23^Medineos Observational Research - An IQVIA Company, Modena, Italy; ^24^Medical Evidence R&I, AstraZeneca, Basiglio, MI, Italy; ^25^Medical Affairs R&I, AstraZeneca, Basiglio, MI, Italy; ^26^UOC Pneumologia, Ospedale “S. Valentino,” Montebelluna (TV) - AULSS 2 Marca Trevigiana, Treviso, Italy; ^27^Department of Health Sciences and Pneumology, University of Milan, ASST Papa Giovanni XXIII, Bergamo, Italy

**Keywords:** severe eosinophilic asthma, switch, benralizumab, observational, biologics

## Abstract

**Background:**

Severe asthma is a heterogeneous inflammatory disease driven by eosinophilic inflammation in the majority of cases. Despite biologic therapy patients may still be sub-optimally controlled, and the choice of the best biologic is a matter of debate. Indeed, switching between biologics is common, but no official guidelines are available and real-world data are limited.

**Materials and methods:**

In this *post hoc* analysis of the Italian, multi-center, observational, retrospective study, ANANKE. Patients with severe eosinophilic asthma treated with benralizumab were divided in two groups based on history of previous biologic therapy (biologic-experienced [suboptimal response] vs naïve). Baseline clinical and laboratory characteristics were collected in the 12 months prior to benralizumab treatment. Change over time in blood eosinophils, annualized exacerbation rate (AER), asthma control (ACT), lung function and oral corticosteroid (OCS) use following benralizumab initiation were collected in the two groups.

**Results:**

A total of 147 biologic-naïve and 58 biologic-experienced (34 omalizumab, 19 mepolizumab, and 5 omalizumab-mepolizumab) patients were enrolled. Biologic-experienced patients were more likely to be atopic and have a higher AER despite more frequent OCS use. Similar reductions in AER (>90% in both groups), OCS use (≥49% reduction in dosage and ≥41% able to eliminate OCS), ACT improvement (≥7 points gained in 48 weeks) and lung function (≥300 mL of FEV_1_ improvement in 48 weeks) were observed after benralizumab introduction within the two groups. There were no registered discontinuations of benralizumab for safety reasons.

**Conclusion:**

In this *post hoc* analysis, patients who were switched to benralizumab because of suboptimal control with a previous biologic therapy were more likely to be atopic and more often treated with omalizumab. Benralizumab is effective in both naïve patients and those previously treated with a biologic.

## Introduction

Severe asthma is a complex and heterogeneous disease that affects 5–10% of patients with asthma (1), and is characterized by the presence of severe exacerbations, systemic corticosteroid use and costs related to healthcare resource utilization (2).

To date, five monoclonal antibodies have been approved for the treatment of asthma, namely omalizumab, mepolizumab, reslizumab, benralizumab, and dupilumab. Omalizumab was the first biologic drug approved for severe allergic asthma, defined by elevation of total serum IgE and perennial allergen sensitization (3). Mepolizumab, reslizumab, benralizumab, and dupilumab have since been approved for severe eosinophilic asthma (SEA), one of the most frequent, severe and difficult-to-treat asthma subtypes (4, 5).

Patients are often eligible for multiple biological treatments because of overlapping characteristics (e.g., eosinophilia and sensitization to a perennial allergen). There is a lack of clear guidelines on how to prioritize the best biologics in patients meeting multiple prescribing criteria (6), direct head-to-head comparison trials of all biologics are not available, and indirect meta-analysis has shown discordant non-conclusive results (7). Presence of specific clinical characteristics such as CRSwNP or allergic rhinitis may help clarify patient phenotype and predict clinical response to biologics (8–10). In line with these evidences, a recent ANANKE *post hoc* analysis confirmed clinical trial data which indicated CRSwNP as the cardinal predictor of benralizumab response (11).

For all of these reasons, the choice of which biologic to prescribe should be individualized for each patient on the basis of many factors, including asthma severity, phenotype, endotype, safety, costs, and expected treatment goals (12).

Once initiated, the effectiveness of a biologic should be evaluated after 4–6 months of treatment in terms of asthma control, exacerbation history, lung function, and other metrics (13). Suboptimal response should ideally be defined as a composite outcome as the reduction of exacerbation rates may not be apparent for more than 1 year after the introduction of a biologic (14). A recent US study using claims data from patients with severe asthma treated with biologics (*N* = 3,262) showed that roughly 60% of patients were uncontrolled or suboptimally controlled despite biologic treatment (15). In this subgroup of patients, switching from one biologic to another should be carefully evaluated according to characteristics related to disease- (e.g., presence of autoimmunity, elevated eosinophils, and fractional exhaled nitric oxide [FeNO]) and patient-related factors (13, 16).

This *post hoc* analysis from the ANANKE (chAracterization of ItaliaN severe uncontrolled Asthmatic patieNts Key features when receiving benralizumab in a real-life setting: the observational rEtrospective) study aimed to describe the clinical characteristics and efficacy of benralizumab in terms of asthma exacerbation, asthma control, lung function and oral corticosteroid (OCS) use in patients with severe eosinophilic asthma who were biologic treatment-naïve compared to patients switched from other biologics (omalizumab and mepolizumab) because of suboptimal response (biologic experienced).

## Materials and methods

### Study design

The design of the ANANKE study has previously been described (17). In brief, ANANKE (ClinicalTrials.gov Identifier: NCT04272463) is an Italian multi-center, observational, retrospective, cohort study including patients with SEA who started benralizumab therapy as per clinical practice or within the Italian Sampling Program that was activated following benralizumab approval in January 2018 and before reimbursement. Patients were consecutively enrolled between December 2019 and July 2020 at 21 Italian sites. As per the protocol, data collection covered a period of >15 months, i.e., 12 months prior to the index date (initiation of the treatment with benralizumab within the sampling program or per clinical practice) to retrieve a restricted set of clinical data plus at least 3 months between the index date and the enrollment visit. ANANKE was performed in accordance with the principles of the Declaration of Helsinki and the regulations and guidelines governing medical practice and ethics in Italy. Ethical approval was provided by the ethics committees/institutional review boards at each participating site. Each patient signed the informed consent and privacy form. Data were collected from each hospital medical charts according to clinical practice and were entered into the electronic case report form.

### Study population

The study included patients aged ≥ 18 years at the index date with SEA requiring stable treatment with high doses of inhaled corticosteroids and a long-acting β_2_-agonist ± additional asthma controller (e.g., LAMA, LTRA, OCS, according to clinicians’ judgment); and who had started benralizumab, receiving at least one injection ≥3 months before enrollment, with hospital medical charts available from the index date. Patients were excluded if during the observation period they received benralizumab in a clinical trial, or participated in studies imposing a specific patient management strategy which did not correspond to the site’s normal clinical practice.

Patients were stratified into two groups: the first group, defined as “naïve,” included patients who previously had not received an asthma biologic treatment; the second group, “biologic-experienced,” comprised patients who had switched from one or more previous biologics because of uncontrolled disease.

### Outcomes and variables

The primary objective was to describe the clinical features of naïve and biologic-experienced patients as recorded at the index date and during the 12 months prior to benralizumab treatment. Demographics (age, sex, body mass index [BMI], comorbidities, and smoking status), asthma features (age at diagnosis and duration), laboratory features (blood eosinophil count [BEC] and total serum immunoglobulin E [IgE]), and atopic status (defined as the presence of a perennial allergen sensitization demonstrated by skin prick test) were recorded. Lung function parameters, asthma control [defined by Asthma Control Test [ACT] (18)], and OCS use and dosage were also measured. Exacerbations were analyzed according to annualized exacerbation rates for any exacerbation (defined as a physician diagnosed clinically relevant asthma exacerbation) and severe exacerbations [defined as worsening of asthma that lead to one of the following: (a) use of systemic corticosteroids for 3 days or more or a temporary increase in a stable, background dosage of oral corticosteroids; (b) an emergency department or urgent care visit (<24 h) due to asthma that required systemic corticosteroids; or (c) an inpatient admission to hospital (≥24 h) due to asthma].

The secondary objective was to describe clinical outcomes assessed during benralizumab treatment between the index date and end of observation; when available, data at 16, 24, and 48 weeks after the index date were described. Outcomes included: (1) change over time of BEC; (2) annualized rate of any exacerbation and severe exacerbations during benralizumab treatment; (3) change over time of asthma control; (4) change over time of FEV_1_; (5) change over time of OCS use and dosage; (6) benralizumab discontinuation and reasons for discontinuation during the observation period. These outcomes were collected and compared in naïve and biologic-experienced patients.

### Statistical analysis

The statistical analysis has previously been described (17). In brief, the analyses were descriptive and carried out using mean, standard deviation (SD), median, interquartile range (IQR), range, and absolute and relative frequencies. No formal hypotheses were pre-specified. The analyses were performed using SAS software v9.4 (SAS Institute, Cary, NC, United States).

## Results

### Study population

Between December 2019 and July 2020, a total of 205 patients were recruited and met the eligibility criteria for the ANANKE study. A total of 147 (71.7%) naïve and 58 (28.3%) biologic-experienced patients were considered evaluable for this *post hoc* analysis (Table 1). Thirty-four of these patients had been previously treated with omalizumab (58.6%), 19 patients (32.8%) with mepolizumab and 5 patients (8.6%) with omalizumab followed by mepolizumab.

**TABLE 1:** Demographics, clinical, and laboratory features of the patient population.

Characteristics at index date	Total population (*N* = 205)	Biologic-naïve (*N* = 147)	Biologic-experienced (*N* = 58)
Female, *n* (%) (*N* = 205)	126 (61.5%)	97 (66.0%)	29 (50%)
Mean (SD) age, years (*N* = 205)	55.8 (13.3)	56.5 (12.7)	53.9 (14.5)
Mean (SD) age at diagnosis of asthma, years (*N* = 203, 110, 91)	38.9 (16.7)	39.9 (17.3)	36.2 (14.1)
Median (IQR) duration of asthma, years (*N* = 203)	12.4 (6.3–24.6)	11.9 (6.3–24.6)	14.6 (6.8–26.5)
Median (IQR) blood eosinophil count (cells/mm^3^)	580 (400–850)	618 (440–915)	500 (300–719)
Median (IQR) total serum IgE, IU/mL (*N* = 123, 60, 61)	289 (85–573)	214 (72.4–476.3)	354.4 (168–620)
Atopy, *n* (%) (*N* = 205)	85 (41.5%)	51 (34.7%)	34 (58.6%)
Positive history of nasal polyposis, *n* (%)	110 (53.7%)	82 (55.8%)	28 (48.3%)
BMI status, *n* (%) (*N* = 205)			
Under/Normal	70 (34.4%)	56 (38.1%)	14 (24.1%)
Overweight	79 (38.5%)	53 (36.1%)	26 (44.8%)
Obese	33 (16.1%)	23 (15.6%)	10 (17.2%)
Unknown	23 (11.2%)	15 (10.1%)	8 (13.8%)
Smoking status, *n* (%) (*N* = 205)			
Non-smoker	139 (67.8%)	103 (70.1%)	36 (62.1%)
Previous smoker	59 (24.4%)	33 (22.4%)	17 (29.3%)
Current smoker	6 (2.9%)	5 (3.4%)	1 (1.7%)
Unknown	10 (4.9%)	6 (4.1%)	4 (6.9%)
Mean (SD) pre-bronchodilator FEV_1_, L (*N* = 154, 74, 70)	2.0 (0.8)	2.0 (0.8)	2.0 (0.8)
Mean (SD) pre-bronchodilator FEV_1_,% predicted (*N* = 159, 79, 71)	70.6 (21.6)	71.2 (21.6)	69.3 (22.9)
Mean (SD) post-bronchodilator FEV_1_, L (*N* = 92, 47, 41)	2.1 (0.9)	2.1 (0.8)	2.2 (1.0)
Mean (SD) post-bronchodilator FEV_1_,% predicted (*N* = 90, 46, 40)	75.3 (22.9)	76.6 (20.2)	71.1 (29.7)
Mean (SD) pre-bronchodilator FVC, L (*N* = 148, 71, 67)	3.0 (1.0)	2.9 (1.0)	3.0 (1.0)
Mean (SD) pre-bronchodilator FEV_1_/FVC (*N* = 148, 71, 67)	0.7 (0.1)	0.7 (0.2)	0.6 (0.1)
Mean (SD) ACT score (*N* = 161, 91, 70)	14.7 (4.7)	14.7 (4.5)	14.8 (5.1)
OCS use, *n* (%)	53 (25.8%)	33 (22.4%)	20 (34.5%)
Mean (SD) OCS dose at index date (*N* = 48, 30, 18)	14.0 (10.3)	11.3 (8.4)	18.0 (11.9)
Annualized exacerbation rate (*N* = 195, 140, 55)	4.03	3.91	4.34
Annualized severe exacerbation rate (*N* = 195, 140, 55)	1.10	0.83	1.79

BMI, body mass index; FEV_1_, forced expiratory volume in the first second; FVC, forced vital capacity; ACT, asthma control test; OCS, oral corticosteroids; SD, standard deviation; IQR, interquartile range (25–75%).

### Clinical characteristics

The proportion of male and female patients was well balanced in the biologic-experienced group but females were more common in the naïve group (50 vs. 66%, respectively). Duration of asthma from time of diagnosis was longer in biologic-experienced patients. As expected from a higher number of patients treated with omalizumab before benralizumab, biologic-experienced patients were more likely to be atopic (58.6 vs. 34.7% in the naïve group) with numerically higher serum IgE levels at baseline. As expected, BEC at baseline was lower in the biologic-experienced group (median 500 cells/mm^3^, IQR = 300–719) when compared to naïve patients (618 cells/mm^3^, IQR = 440–915). The two groups were otherwise comparable in terms of age, age at diagnosis of asthma, BMI and smoking status.

Lung function based on FEV_1_, FVC, and FEV_1_/FVC as well as asthma control (ACT) were impaired (under the normal range) in both groups. A numerically higher rate of any exacerbation (4.34 vs. 3.91 in the naïve patients) and severe exacerbations (1.79 vs. 0.83 in the naïve patients) were present in the biologic-experienced group. A slightly higher percentage of patients in the biologic-experienced group were treated with OCS before benralizumab start (34.5 vs. 22.4% in the naïve group), with a higher prednisone-equivalent daily dosage (mean of 18 vs. 11.3 mg/day in the naïve group). Ten patients in the naïve group (7.1%) and four patients in the biologic-experienced group (7.2%) did not show any exacerbation during the 12 months before benralizumab prescription.

Comorbidities considered to be possibly related to OCS use, such as hypertension, osteoporosis, cataract, anxiety/depression, type 2 diabetes mellitus and cardiovascular disease were numerically more prevalent in the biologic-experienced group when compared to the naïve group (Table 2).

**TABLE 2:** List and percentage of comorbidities within the study population.

	Total population (*N* = 205)	Biologic-naïve (*N* = 147)	Biologic-experienced (*N* = 58)
≥1 Relevant comorbidity	175 (85.4%)	124 (84.4%)	51 (87.9%)
≥1 Current asthma-related condition	103 (50.2%)	75 (51.0%)	28 (48.3%)
GERD	43 (21%)	30 (20.4%)	13 (22.4%)
Allergic conjunctivitis	28 (13.7%)	23 (15.6%)	5 (8.6%)
Allergic rhinitis	45 (22%)	35 (23.8%)	10 (17.2%)
≥1 Current OCS-related condition	77 (37.6%)	49 (33.3%)	28 (48.3%)
Hypertension	46 (22.4%)	29 (19.7%)	17 (29.3%)
Osteoporosis	23 (11.2%)	14 (9.5%)	9 (15.5%)
Cataract	12 (5.9%)	7 (4.8%)	5 (8.6%)
Anxiety/depression	11 (5.3%)	4 (2.7%)	7 (12%)
Type 2 diabetes mellitus	10 (4.9%)	5 (3.4%)	5 (8.6%)
Obstructive sleep apnea	10 (4.9%)	6 (4.1%)	4 (6.9%)
Cardiovascular disease	7 (3.4%)	3 (2.0%)	4 (6.9%)
≥1 Other ongoing comorbidities	35 (17.1%)	23 (15.6%)	12 (20.7%)

GERD, gastoesophageal reflux disease.

Both groups had a mean ± SD exposure to benralizumab therapy of 10.3 ± 5.0 months and a median [IQR] exposure of 9.8 [6.1–13.9] months after index date.

### Effect of benralizumab on blood eosinophil count

A near complete depletion of peripheral eosinophils was seen in both groups at the first timepoint (16 weeks), and BEC remained low thereafter, in accordance with the known mechanism of action of benralizumab (Supplementary Figure 1).

### Effect of benralizumab on exacerbations rates

**FIGURE 1:**
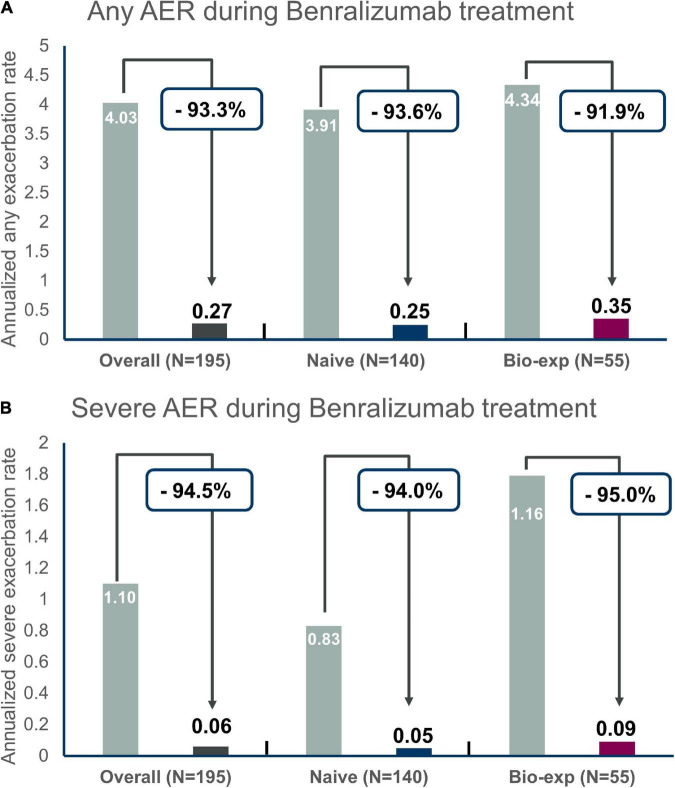
Annualized exacerbation rates (AER) of any severity **(A)** and for severe exacerbations **(B)** during benralizumab treatment in the entire population and in naïve severe eosinophilic asthma patients versus biologic-experienced patients.

The annualized exacerbation rate was consistently reduced after the introduction of benralizumab in both groups (−93.6% reduction in naïve patients and −91.9% reduction in biologic-experienced patients) (Figure 1A). In particular, during the observation period, the percentage of patients without any exacerbations increased from 7.1 to 82.4% in the naïve group and from 7.2 to 80% in the biologic-experienced group of patients. Benralizumab reduced the severe annualized exacerbation rate in naïve and biologic-experienced patients by −94 and −95%, respectively (Figure 1B), and there were no differences between the two groups. At index date biologics-experienced patients seemed more severe than naïve patients as they showed trend toward higher AER, OCS use and lower FEV1.

### Effect of benralizumab on asthma control

**FIGURE 2:**
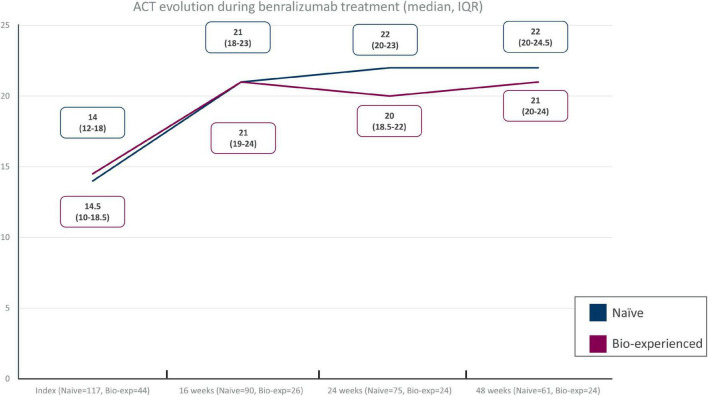
Asthma control test (ACT) improvement in different timepoints in severe eosinophilic asthma (SEA) patients without (naïve) and with (biologic-experienced) previous use of a biologic drug during benralizumab treatment.

Improvements in ACT were seen as early as at the first timepoint (16 weeks), reaching a median of 21 points in both groups. The improvement was sustained throughout the other timepoints (24 and 48 weeks) both in naïve (22 points, IQR = 20–24.5) and biologic-experienced patients (21 points, IQR = 20–24 points) (Figure 2). A total of 82% of the naïve patients scored at least 20 points and 77.8% achieved the minimal important difference (MID) of ACT at 48 weeks. Similarly, 79.2% of the biologic-experienced patients presented an ACT of at least 20 points and 73.7% achieved an MID of ACT at Week 48.

### Effect of benralizumab on lung function

**FIGURE 3:**
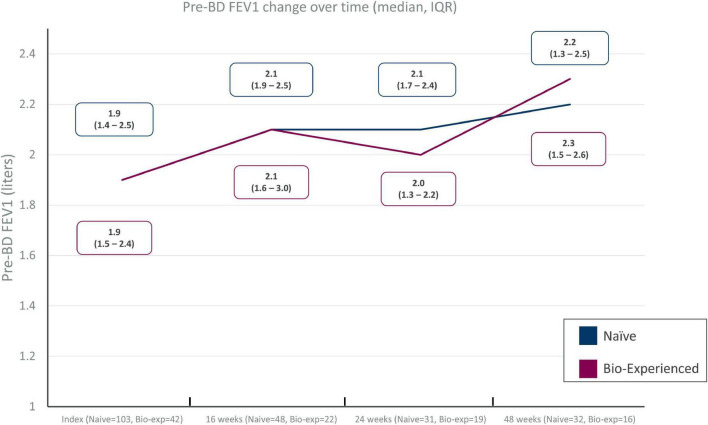
Pre-BD FEV_1_ change over time in severe eosinophilic asthma (SEA) patients with (biologic-experienced) and without (naïve) previous exposure to a biologic drug during benralizumab treatment.

Sufficient data for the evaluation of lung function were only available for pre-bronchodilator FEV_1_ at 16, 24, and 48 weeks during benralizumab treatment in the two groups. Improvement of FEV_1_ was evident in both groups, with +200 mL gain after 16 weeks of treatment and +400 and +300 mL gain at 48 weeks in the naïve and biologic-experienced group, respectively (Figure 3). Data are not shown for the other lung function parameters described at baseline.

### Steroid-sparing effect of benralizumab

**FIGURE 4:**
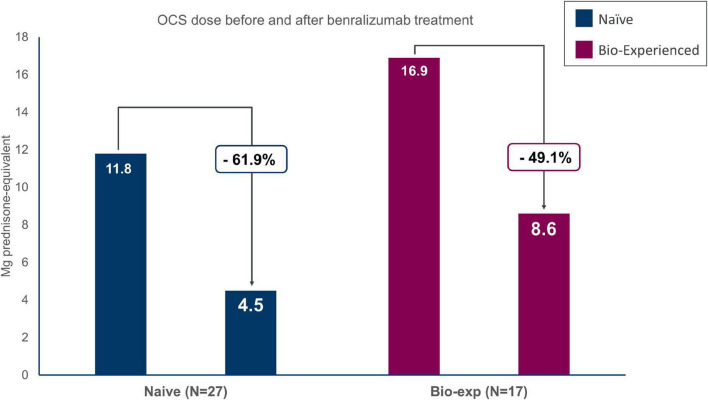
Oral corticosteroids (OCS) sparing effect of benralizumab in severe eosinophilic asthma (SEA) patients with (biologic-experienced) and without (naïve) previous use of a biologic drug. Dose is reported in milligrams of prednisone equivalent.

Data on OCS reduction and elimination during benralizumab treatment was available for 27 out of 33 naïve patients and 17 out of 20 biologic-experienced patients with OCS use at index date (Figure 4 and Table 3).

**TABLE 3:** Oral corticosteroid (OCS) reduction at end of observation in the patient population.

	Biologic-naïve (*N* = 27)	Biologic-experienced (*N* = 17)
**Reduction from baseline, *n* (%)**		
100%	11 (40.7%)	8 (47.1%)
≥90%	11 (40.7%)	8 (47.1%)
≥75%	12 (44.4%)	8 (47.1%)
≥25%	13 (48.1%)	8 (47.1%)
Anyreduction, *n* (%)	13 (48.1%)	9 (52.9%)
No reduction, *n* (%)	14 (51.9%)	8 (47.1%)

Naïve patients reduced OCS dosage by 61.9%, decreasing from 11.8 ± 8.5 to 4.5 ± 5.6 mg/day of prednisone-equivalent at the end of the observation period. Forty-one percent of the patients were able to eliminate OCS and 48.1% obtained any reduction of the OCS dosage. Similarly, biologic-experienced patients reduced OCS dose from 16.9 ± 9.1 to 8.6 ± 10.3 mg/day of prednisone-equivalent (−49.1%); 47.1% of the patients being able to completely eliminate OCS at EOB.

### Discontinuation and safety

Three patients in the naïve group and one patient in the biologic-experienced group discontinued benralizumab during the observation period. Lack of efficacy, physician, or patient decision were the reasons recorded for discontinuation. No discontinuation for safety reasons were registered after index date.

## Discussion

In this *post hoc* analysis of the real-world ANANKE study (17) we evaluated the clinical characteristics of 205 SEA patients who were either biologic-naïve or switched from another biologic therapy because of suboptimal response. We confirmed the efficacy of benralizumab in all the outcomes evaluated, even in patients who failed a previous biologic therapy (omalizumab or mepolizumab). Benralizumab showed a reduction of over 90% in asthma exacerbations (even severe exacerbations), a reduction in concomitant OCS use (with almost 50% of patients having elimination of OCS), as well as improvements in asthma control amelioration and lung function independent of previous biologic therapy failure.

In this study, nearly 30% of the patients were biologic-experienced at the time of benralizumab prescription, a percentage similar to that recently described in the literature (19–21). More than half of the patients in this study switched from omalizumab to benralizumab, and 8.6% were eligible for omalizumab before switching to mepolizumab and then to benralizumab because of lack of efficacy. Despite previous treatment with omalizumab or mepolizumab, the subgroup of biologic-experienced patients seemed more severe than biologics naïve as they presented higher AER, more OCS dependence and lower FEV1. As expected, the presence of atopy, defined as the presence of sensitization to a perennial allergen along with serum IgE levels (17), was numerically more frequent in the biologic-experienced population. To date, no specific biomarker has been proven to be useful for predicting response to omalizumab in severe allergic asthmatic patients (22). In contrast, eosinophil counts of ≥300 cells/mm^3^, the presence of nasal polyps, basal corticosteroid use and a late onset of asthma have been found to predict an enhanced response to benralizumab (23).

Switching from omalizumab to an anti-interleukin (IL)-5 biologic therapy has been proven efficacious in ameliorating exacerbation rates and OCS use, as well as improving asthma control and lung function in severe allergic asthma patients with an eosinophilic phenotype (6, 24–28). To date, only one real-world study has been published on the efficacy of benralizumab in severe allergic asthma patients with a BEC of ≥300 cells/mm^3^ and who had previously received omalizumab (29). In particular, benralizumab was shown to significantly reduce asthma exacerbation rates and OCS use, with parallel improvement in asthma symptom control and lung function, not only versus baseline, but also with respect to omalizumab treatment. These results are most likely related to the specific mechanism of action of benralizumab – antibody-dependent cellular cytotoxicity (ADCC) – which allows almost complete elimination of peripheral blood and tissue eosinophils (30). A direct correlation between eosinophil levels and risk of exacerbations has been observed in allergic patients who had eosinophils levels of 300 cells/mm^3^ or higher (31). In contrast, no association between serum IgE levels and risk of exacerbation or other asthma outcomes has been evidenced so far (31).

Nineteen patients in this study switched to benralizumab from mepolizumab due to suboptimal control. To date, limited information is available regarding the effectiveness of switching between agents targeting the anti-IL-5/anti-IL-5R pathway (32). A retrospective study by Jackson et al. (33) demonstrated that benralizumab was able to reduce exacerbation rates and OCS use while improving asthma control and lung function in patients sub-optimally controlled with mepolizumab therapy. One-third of the patients did not experience any exacerbations after 1 year of benralizumab treatment, and more than half of the patients completely eliminated OCS use. The limited number of patients in our study precludes us from making specific conclusions about these 19 patients. However, the reduction of exacerbation rate by over 90% is commensurate with the efficacy of benralizumab even in this subgroup of patients.

To date, no clinical differences between responders and non-responders to mepolizumab have been identified that predict subsequent response to benralizumab (33). Three main reasons can potentially explain the efficacy of benralizumab in patients who have failed an anti-IL-5 drug such as mepolizumab. Firstly, benralizumab is the only drug that is able to achieve a near complete depletion of eosinophils both in peripheral blood and tissues, including the airways (30, 34, 35). The phenotyping Mepolizumab EXacerbations in severe eosinophilic asthma (MEX) study revealed that approximately 50% of the exacerbations in patients treated with mepolizumab were eosinophilic in nature, showing persistence of sputum eosinophil levels ≥2% after treatment (36). In these sub-optimally controlled patients, identified by increasing FeNO levels during exacerbations (36), benralizumab should be considered, even during the phenotyping process in biologic-naïve patients (37). Alternatively, switching to anti-IL-4/IL-13 could be considered in this subgroup of patients, but careful monitoring of BEC and clinical parameters are recommended to assess the risk of symptomatic hypereosinophilia and/or evolution toward eosinophilic granulomatosis with polyangiitis (EGPA, former known as Churg-Strauss Syndrome) (38, 39). Secondly, autoimmune features may be present in severe asthma patients (40). In particular, IL-5-anti-IL-5 complement activating immune-complexes and anti-eosinophils peroxidase have been reported to reduce the effectiveness of mepolizumab fixed dose (100 mg every 4 months), but not intravenous mepolizumab 750 mg, reslizumab 3 mg/kg or benralizumab 30 mg every 8 weeks (12, 13, 16, 40, 41). Finally, switching to benralizumab from another biologic of the anti-IL-5 pathway can be adequate when the presence of anti-drug autoantibodies (ADA) has been suspected as the cause of failure (13). No clear factors have been associated with higher ADA risk and, to date, intermittent biologic therapy and re-exposure after a long treatment-free interval may be associated with higher ADA risk (42). The presence of ADA has not been assessed in our study, as a limited number of Centers offer this possibility in real-life.

Few reports of double switching from omalizumab to mepolizumab and then benralizumab are reported in the literature (43). In our study, five patients switched from omalizumab to mepolizumab and then to benralizumab for suboptimal response to the first two biologics. Further analyses are planned for this special subgroup of patients and specific data are not shown in this study because of limited long-term information (e.g., ACT score and FEV_1_ at 48 weeks).

The use of OCS in SEA patients deserves specific commentary (44). In this study, half of the patients who switched from another biologic eliminated OCS use and an overall reduction of 50% in OCS from the baseline dose was achieved in less than 1 year of follow-up. The particular pharmacodynamic characteristics of benralizumab, such as the unique mechanism of action (near complete eosinophil depletion through ADCC), the action on precursors of eosinophils (45) and the more prominent suppression of the IL-5 axis (46) may account for these effects on patients who failed treatment with omalizumab and mepolizumab.

This study does have several limitations, some of which have already been discussed above. We acknowledge the retrospective nature of the study and the absence of a comparator arm may be limiting, as well as the absence of baseline clinical and laboratory data for switched patients before the introduction of the first biologic.

Moreover, the observational nature of the study did not allow to make any formal statistical hypotheses and the results are descriptive only. However, we think that this study represents the real-world experience on the use of benralizumab in both naïve and biologic-experienced patients, and we believe it could be informative for clinicians in daily clinical practice (47).

## Conclusion

This study described the clinical features of patients switching from other biologics to benralizumab, with a numerically higher prevalence of atopy related to the frequency of omalizumab use in our population. Benralizumab has been shown to be effective in naïve patients and biologic-experienced patients in reducing asthma exacerbation and OCS use and improving lung function and asthma control. Previous use of mepolizumab should not be a deterrent for the use of benralizumab in suboptimal responders, likely due to differences in the mechanism of action between the two drugs. Further studies characterizing the clinical profile of patients benefiting the most from biologics in severe asthma are warranted in order to avoid multiple switches between biologics.

## Data availability statement

The datasets used and/or analyzed during the current study are available from the corresponding author on reasonable request.

## Ethics statement

The ethical approval was provided by the Ethics Committees/Institutional Review Boards at each participating site. The patients/participants provided their written informed consent to participate in this study.

## Author contributions

FM, PC, MA, PB, LB, MFC, CC, SC, MD’A, SD, FDi, FDe, EP, GP, PR, MR, PS, MC, AV, and GC: investigation, resources, and writing – review and editing. AZ and SR: methodology, software, validation, formal analysis, resources, data curation, and writing – review and editing. SB: conceptualization, writing – original draft, writing – review and editing, and project administration. GV: conceptualization, writing – original draft, writing – review and editing, and visualization. All authors contributed to the article and approved the submitted version.
