# Supplementary material for: Switching from one biologic to benralizumab in patients with severe eosinophilic asthma: An ANANKE study post hoc analysis
Source: Front Med (Lausanne). 2022 Sep 2;9:950883. doi: 10.3389/fmed.2022.950883 (PMC9478391; doi:10.3389/fmed.2022.950883)
Supplement: Supplementary file 1 [file Table_1.DOCX]

Supplementary Material

## Supplementary Figures

**
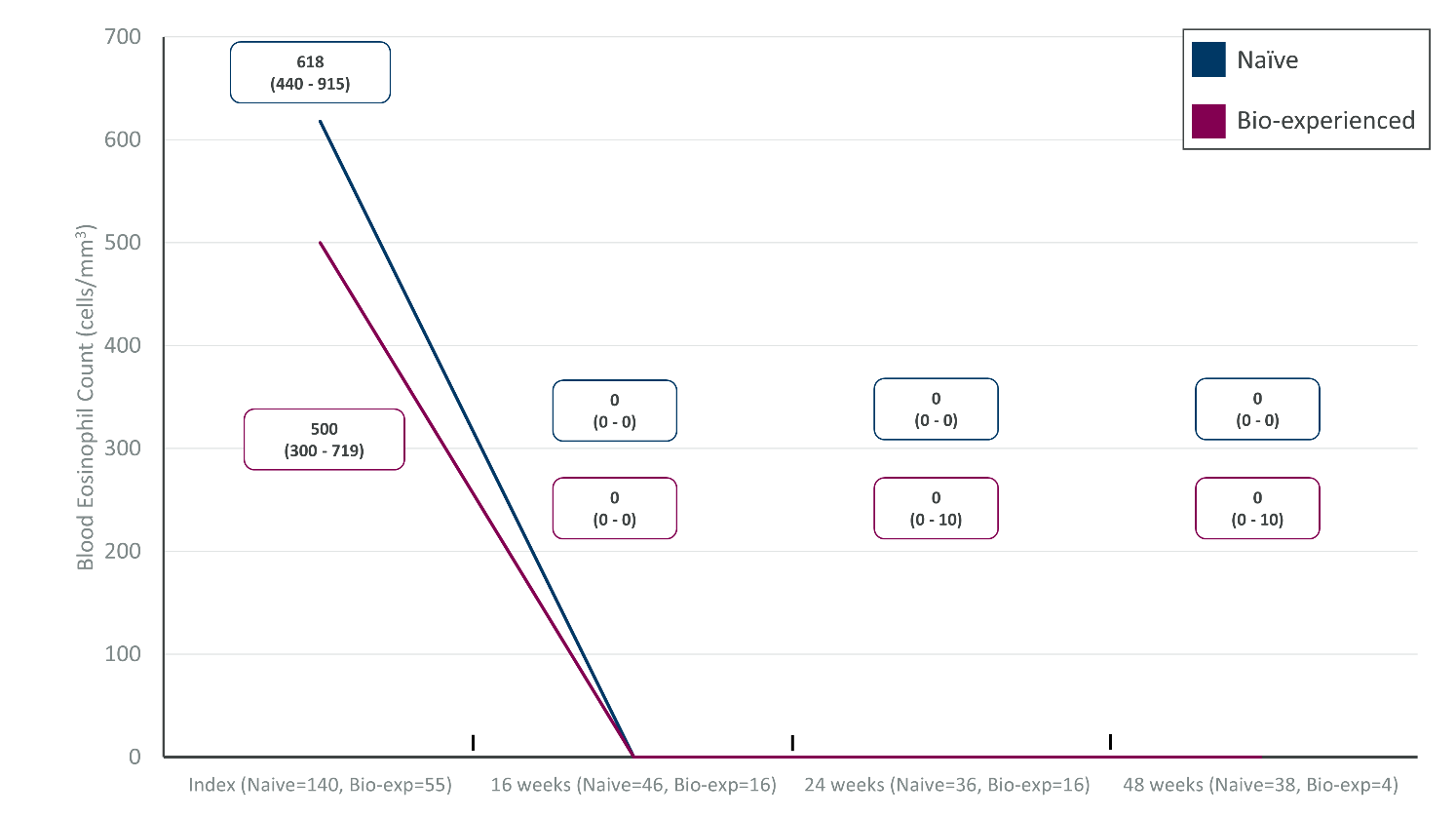
**

**Supplementary Figure 1.** Effect of benralizumab on blood eosinophil count at different timepoints.
